# Nonfacial Use of Injectable Poly-L-lactic Acid: Safety Data From a Multicenter Observational Study in the United States

**DOI:** 10.1093/asjof/ojag053

**Published:** 2026-03-25

**Authors:** Kalpna Kay Durairaj, Ali Vafa, Shino Bay Aguilera, Sabrina G Fabi, Neil Sadick, Melanie D Palm, Jennifer Levine, Sachin M Shridharani, Flor Mayoral, Daniel Bråsäter, Inna Prygova, Charlotta Wolgast, Felipe Weinberg

## Abstract

**Background:**

Poly-L-lactic acid (PLLA-SCA) was approved for facial aesthetic use in the USA in 2009. Nonfacial aesthetic treatment using PLLA-SCA, a collagen-stimulating injectable implant, is a frequent clinical practice, and initial studies support its safety.

**Objectives:**

The objective of this study was to evaluate the safety of PLLA-SCA when used in nonfacial areas in real clinical practice in the USA.

**Methods:**

In this retrospective chart review, subjects treated in a nonfacial area with ≥2 vials of PLLA-SCA in ≥2 treatment sessions were eligible. Medical charts from 9 US sites were reviewed to collect demographics, treatment details, and treatment-related adverse events (AEs). The primary endpoint was treatment-related AEs.

**Results:**

Among the 498 subjects included, most subjects (72%) had 2 or 3 treatment sessions, with an average of 4.0 vials/treatment session. Reconstitution volumes were generally ≥10 mL/vial for all treatment areas, with 47% of treatments using volumes of 10-15 mL, and another 47% using ≥15 mL/vial. The most common treatment area was the gluteal region (buttocks or hip dip; 60.3% of sessions) and decolletage (8.2% of sessions). Most treatment-related AEs were injection related and mild or moderate in intensity. The most common AE was injection site bruising (5.0%), and AEs were mainly reported in the buttocks (4.0%) or posterior thigh (1.0%). Nodule occurrence was low (1 subject, 0.2%).

**Conclusions:**

Poly-L-lactic acid was well tolerated in real-world use for treatment of a range of nonfacial areas, with typically larger volumes than usually used for facial treatment.

**Level of Evidence: 3 (Therapeutic):**

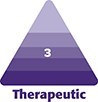

Sculptra^®^ poly-L-lactic acid (Galderma, Zug, Switzerland), hereafter, PLLA-SCA, is a collagen-stimulating injectable implant with regenerative properties, including adipocyte regeneration and elastin stimulation, through a combination of mechanotransduction (response from cells to physical forces) and foreign body reactions.^1–3^ PLLA-SCA was first US Food and Drug Administration (FDA)-approved for restoration and correction of facial fat loss (ie, lipoatrophy) in people with HIV in 2004 and is indicated since 2009 in the USA for use in the correction of nasolabial fold contour deficiencies.^4, 5^ Recently, it has also been approved in the US for correction of fine lines and wrinkles in the cheek region and in China for midface volume deficit and midface contour deficiency.

Similar to rejuvenating the facial area, patients seek rejuvenating treatments in nonfacial/body areas to align how they look with how they feel. PLLA-SCA is known to be used for aesthetic treatment of nonfacial areas, for example, in the buttocks, hands, neck, decolletage wrinkles, thighs, and knees, as well as for laxity of the medial upper arm, periumbilical, and abdominal areas^6–10^; yet, scarce studies and publications exist for these treatment areas.^11^ Use of PLLA-SCA to treat any area of the body should be at the injector's discretion, taking into account that regulatory approvals may vary between geographic regions.

The safety of PLLA-SCA used in nonfacial indications has been demonstrated in previous studies. Clinical trials suggest that the product is well tolerated: 2 randomized controlled trials (RCT) in knee laxity^12^ and lower extremity cellulite,^13^ and 3 open-label trials in treatment of the buttock area,^14^ decolletage,^15^ and skin sagging of the gluteal region.^16^ The objective of the current retrospective study was to evaluate the safety of PLLA-SCA when used in nonfacial areas in real clinical practice in the USA.

## METHODS

### Study Design and Subjects

This multicenter, retrospective medical chart review (NCT05463978) was performed at investigational sites with experience in PLLA-SCA treatment (that treated over 30 subjects with PLLA-SCA from January 2018 to December 2020). Data collection commenced 18 months following this period to ensure that any reported late onset adverse events would be captured from the medical charts. The study was conducted in compliance with Good Clinical Practice guidelines and applicable regional and national regulations and conformed to the Declaration of Helsinki. Information was retrieved from medical charts, and no study visits were performed by the subjects.

Eligible subjects had previously been injected with PLLA-SCA in nonfacial areas as specified hereafter. Subjects could be excluded at the discretion of the investigator or if they actively asked not to be involved in the study. Concurrent and/or prior treatment with PLLA-SCA in the face was not a reason for exclusion.

### Treatment

Eligible subjects had undergone at least two different treatment sessions using at least two vials of PLLA-SCA each time. Of note, at the time of subject treatment, these indications were noncompliant with the current intended use per PLLA-SCA Instructions for Use in the USA.

### Collected Data

The primary objective was to evaluate the safety of PLLA-SCA when used in nonfacial areas in real clinical practice in the USA. The primary endpoint was adverse events (AEs) identified by the investigator as related to the product or injection procedure reported in the medical chart. Injection details (eg, area treated, the number of vials and reconstitution volume used, and injection depth), demographic data, and history of medical conditions, surgical procedures, and/or nonpharmacological treatments were also recorded.

### Statistical Methods

Descriptive statistics were performed for all variables. Analyses were done on the full analysis set (FAS, ie, subjects meeting all eligibility criteria).

## RESULTS

### Subject Disposition and Demographics

A total of 498 subjects from 9 aesthetic clinical sites in the USA with experience in PLLA-SCA and body treatment were included in the FAS. The majority were female (96.4%), and the mean age was 42.1 years, ranging from 18 to 82 years old (Table 1). The most commonly treated areas were the gluteal region in 60.3% of sessions (buttocks and hip dip areas [51.5% and 8.8%], respectively), decolletage area (8.2%), thighs (6.5%), and above the knees (6.0%) (Table 2). Other areas that were treated (8.1%) included combined areas (ie, buttocks and thighs), labia majora, and abdomen (midline supraumbilical concavity). Nearly three-fourths of subjects (71.9%) received 2 or 3 treatment sessions (mean: 3.2 treatment sessions/subject).

**Table 1.** Demographic Data and Past Medical/Surgical History (FAS)

		Total (*N* = 498)
Age (years)	Mean (SD)	42.1 (11.8)
	Median	42.0
	Min, Max	18, 82
Gender, *n* (%)	Female	480 (96.4)
	Male	17 (3.4)
	Other	1 (0.2)
Ethnicity, *n* (%)	Not Hispanic/Latino	155 (31.1)
	Hispanic/Latino	93 (18.7)
	Not included in medical record	250 (50.2)
Race, *n* (%)^a^	Asian	25 (5.0)
	Black/African American	6 (1.2)
	Native Hawaiian/Other Pacific Islander	2 (0.4)
	White	166 (33.3)
	Other	31 (6.2)
	Multiple^a^	10 (2.0)
	Not included in medical record	258 (51.8)
Medical condition, *n* (%)	Cellulite	20 (4.0)
Previous surgical procedures, *n* (%)	Gluteoplasty	15 (3.0)
	Mammoplasty	9 (1.8)
	Liposuction	5 (1.0)

FAS, full analysis set; *N*, number of subjects in full analysis set, *n*, number of subjects in specific category; SD, standard deviation.

^a^The “Multiple race” category included subjects with more than 1 race selected on the electronic case report form.

**Table 2.** Treatment Areas (FAS)

		Total number of sessions *(N = 1651)* *n (%)*
Number of sessions per treatment area	Buttocks	850 (51.5)
	Hip dip	146 (8.8)
	Decolletage	135 (8.2)
	Above the knee	99 (6.0)
	Neck	72 (4.4)
	Arms	56 (3.4)
	Abdomen	52 (3.1)
	Posterior thigh	45 (2.7)
	Lateral thigh	36 (2.2)
	Anterior thigh	26 (1.6)
	Other^a^	134 (8.1)

FAS, full analysis set; *N*, number of treatment sessions overall, *n*, number of treatment sessions given for a specific category; SD, standard deviation.

^a^“Other” includes treatments of combined areas (ie, buttock and thighs [anterior/lateral/posterior] and thighs (anterior/lateral/posterior) as well as labia majora, midline supraumbilical concavity, etc.

### Injection Data

For the FAS population, almost all injections were done subcutaneously or deep dermal. The majority of injections used a needle (82.7%), and some used cannula (12.3%), or cannula and needle, depending on the treatment area (5.0%). An average number of 4.0 vials were used per treatment, with a total mean of 13.4 vials per subject including all treatment areas. Reconstitution volumes (including reconstitution medium and lidocaine) were generally ≥10 mL for all treatment areas (94% of treatment sessions): 47% of treatments were performed using volumes of 10-15 mL, and 47% using ≥15 mL for reconstitution (Table 3). The reconstitution medium for the product was sterile water for injection in 58.1% of sessions, bacteriostatic water in 33.3% of sessions, and other media in 8.5%. The most common reasons for treatment were improvement of volume (56.2%), tightening of sagging/loose skin/laxity (55.2%), or improvement in appearance of cellulite (50.9%).

**Table 3.** PLLA-SCA Reconstitution Volumes (Including Lidocaine) by Number of Sessions and Treatment Area

	Total volume <10 mL		Total volume ≥10 mL to <15 mL		Total volume ≥15 mL		Total volume Not specified		Total	
	*n*	%	*n*	%	*n*	%	*n*	%	*N*	%
Abdomen	8	15.4	22	42.3	22	42.3	0	0.0	52	100
Above the knee	6	6.1	18	18.2	75	75.8	0	0.0	99	100
Arms	10	17.9	25	44.6	21	37.5	0	0.0	56	100
Buttocks	2	0.2	488	57.4	323	38.0	37	4.4	850	100
Decolletage	17	12.6	27	20.0	91	67.4	0	0.0	135	100
Hip dip	0	0.0	128	87.7	18	12.3	0	0.0	146	100
Neck	3	4.2	7	9.7	62	86.1	0	0.0	72	100
Thigh	4	3.7	18	16.8	82	76.6	3	2.8	107	100
Other	8	6.0	42	31.3	84	62.7	0	0.0	134	100
All areas	58	3.5	775	46.9	778	47.1	40	2.4	1651	100

*N*, total number of sessions; *n*, number of sessions; PLLA-SCA, poly-L-lactic acid injectable implant.

### Safety

**Figure 1.**
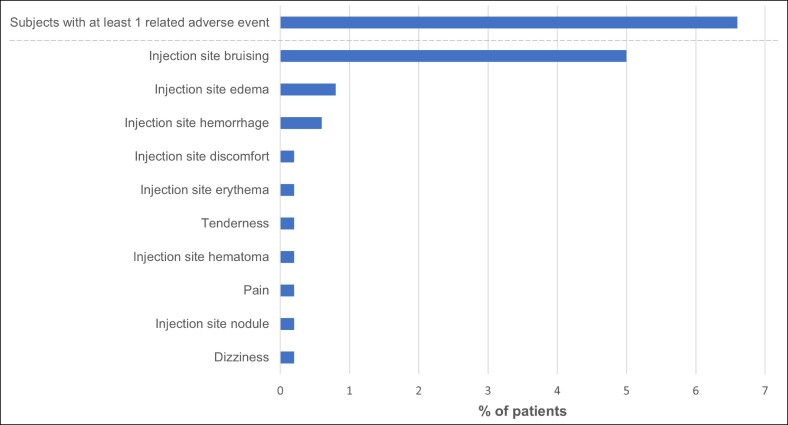
Adverse events judged as treatment-related (overall).

**Figure 2.**
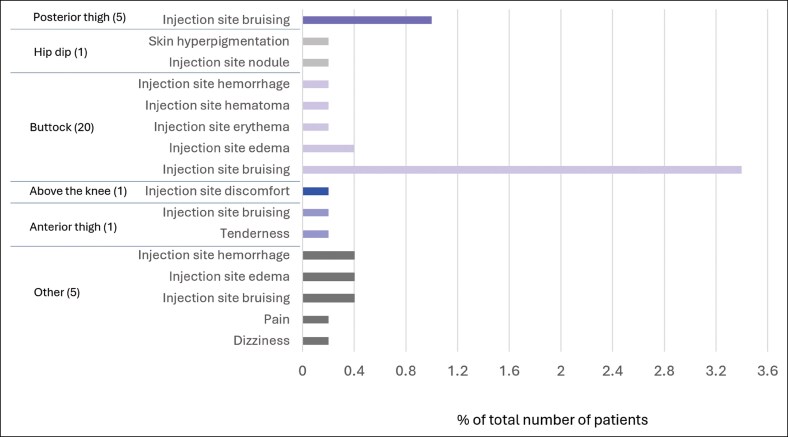
Adverse events by treatment area. The number in parentheses shows the number of subjects reporting treatment-related adverse events.

Treatment with PLLA-SCA for nonfacial indications was overall well tolerated. Treatment-related AEs were mild or moderate and experienced in 6.6% of subjects, most commonly injection site bruising (5%) (Figure 1). Four (0.8%) subjects had moderate events that resolved (injection site bruising, 2 subjects [0.4%]; edema, 1 subject [0.2%]; and nodule/papule in the hip dip, 1 subject [0.2%]). For the majority (79.6%) of events, no action was needed. Treatment-related AEs were observed in the buttocks region of 20 (4%) subjects, posterior thigh of 5 (1%) subjects, and above the knee, anterior thigh, and hip dip areas of 1 (0.2%) subject each (Figure 2). There were no serious AEs or late onset treatment-related AEs (late onset defined as starting >21 days after the most recent treatment session).

## DISCUSSION

In this patient safety paper, we describe the outcomes of this retrospective observational study, which support the safety and tolerability of PLLA-SCA when used in nonfacial areas. The safety profile observed in this study was comparable with that in previous studies of PLLA-SCA used in facial areas where AEs were mild or moderate in intensity and most resolved spontaneously,^5,17–19^ even when used at greater reconstitution and injection volumes.^19,20,21^ In addition, the safety profile observed in this study was similar to that in other studies using PLLA-SCA for nonfacial indications.^12, 13,15, 16,22^

Recommendations on PLLA-SCA use in nonfacial areas are starting to expand, such as a consensus from the Asia-Pacific region encompassing injection volumes for both facial and body treatments, and an international consensus highlighting the need for standardized injection protocols across different body areas.^23,24^ Further studies are needed to better understand the efficacy and durability of PLLA-SCA when injected for body treatments. However, the evidence suggests that injecting PLLA-SCA in nonfacial areas is not associated with increased risk of AEs, despite greater volume of PLLA-SCA administered.^8^ In the current study, the buttock region was the most frequently treated area (in just over half of the treatment sessions). This chart review data resonates with another study (prospective, open-label) conducted with PLLA-SCA, which showed that it is well tolerated and effective in buttock contouring, with the most common subject-reported AEs being pain (mean of 71%) and bruising (mean of 29%).^14^ Bruising was also a frequently reported treatment-related AE in our study. Furthermore, in their prospective study, Nikolis et al highlight that mild AEs following injection (eg, bruising) typically resolve within 7-14 days. Moreover, no papules, nodules, or granulomas were observed at any point during the study duration. Their study used PLLA-SCA diluted in a total volume of 18 mL,^14^ as in the present study, in nearly one-third of subjects. This is similar to the clinical experience shared in a case series completed in Brazil by Sarubi et al,^25^ where PLLA-SCA was used with a dilution of 16-20 mL in gluteal augmentation, with favorable patient outcomes. It is interesting to note that while the minority of injections were performed with cannulas and the majority with needles in this US study, non-US clinicians tend to use needles less often. This variability in technique represents a study limitation; however, it may indicate that PLLA-SCA can be injected with either needle or cannula and have similar outcomes.

In our study, cellulite was one of the main treatment requests. These results support the safe treatment of the appearance of cellulite as found in another RCT where PLLA-SCA, along with subcision, improved the appearance of lower extremity cellulite in adult women and was well tolerated.^13^ Additionally, a recent prospective 12-month pilot study indicates that PLLA-SCA in large volumes (median of 54 mL in each thigh per session) improves the appearance of cellulite in the posterior thighs and is well tolerated.^21^ Furthermore, a clinical retrospective review of 60 patients by Durairaj et al found that those who underwent treatment with approximately 20 vials had softening of cellulite dimpling, improvement in volume, and improved skin texture. All AEs were reported as minor and were injection site–related effects, such as bruising, swelling, and soreness.^26^ Cellulite, particularly in postpubertal women, can be frequently associated with distress and reduced quality of life.^13^ Other treatment options exist but are generally considered ineffective, such as topical therapies^27^ and noninvasive energy-based options, which have often led to temporary results and require repetitive treatments.^28^ PLLA-SCA is a safe and viable treatment option to improve the appearance of cellulite, warranting further study.

Although this study is limited by its retrospective study design, meaning that reported rates of AEs in medical charts may be lower than occurrence captured in prospective clinical trials, its strength is in the large number of charts reviewed across 9 aesthetic clinics in the USA, and that it adds to the currently available evidence exploring nonfacial indications. Additional limitations inherent to the retrospective study design include the variability in the reconstitution medium that was used, treatment/selection bias, lack of uniform treatment protocols, variation in the duration of follow-up, that the injector questionnaire was not a validated measure, and heterogeneity of the studied group.

## CONCLUSIONS

Overall, in this real-world retrospective study, PLLA-SCA was administered across diverse nonfacial treatment areas along with a wide range of reconstitution and total volumes (over 10-15 mL) with an average of 3 sessions and 4 vials per session. This suggests that PLLA-SCA is a versatile option for rejuvenation of the skin in nonfacial treatment areas.
